# Engineered Mesenchymal Stem Cells Expressing CD::UPRT and TRAIL Exhibit Potent Anti-Tumor Effects in Glioblastoma Patient-Derived Organoids

**DOI:** 10.3390/cells15171620

**Published:** 2026-09-06

**Authors:** Dokyeong Kim, Minyoung Park, Junseong Park, Soon A Park, Stephen Ahn, Yeun-Jun Chung

**Affiliations:** 1 Department of Microbiology, College of Medicine, The Catholic University of Korea, Seoul 06591, Republic of Korea; dkkim2908@catholic.ac.kr (D.K.); m.young@catholic.ac.kr (M.P.); 2 Precision Medicine Research Center, College of Medicine, The Catholic University of Korea, Seoul 06591, Republic of Korea; j.p@catholic.ac.kr; 3 Catholic Hematopoietic Stem Cell Bank, College of Medicine, The Catholic University of Korea, Seoul 06591, Republic of Korea; 4 Department of Neurosurgery, St. Vincent’s Hospital, College of Medicine, The Catholic University of Korea, Seoul 06591, Republic of Korea; bobby11@catholic.ac.kr; 5 Department of Biomedicine and Health Sciences, College of Medicine, The Catholic University of Korea, Seoul 06591, Republic of Korea

**Keywords:** glioblastoma, mesenchymal stem cells (MSCs), patient-derived organoids, cytosine deaminase::uracil phosphoribosyltransferase (CD::UPRT), TRAIL

## Abstract

**Highlights:**

**What are the main findings?**

- BM03 markedly reduced viability and invasive behavior in patient-derived GBM organoids (GBOs) while suppressing EMT- and stemness-associated markers.
- BM03 showed enhanced migration and infiltration into GBOs, accompanied by increased apoptosis.

**What are the implications of the main findings?**

- These findings validate the multifaceted anti-tumor activity of BM03 in GBOs that retain clinically relevant tumor heterogeneity.
- The results provide organoid-based preclinical evidence supporting further in vivo evaluation of BM03 as an MSC-based targeted therapeutic strategy for GBM.

**Abstract:**

Glioblastoma (GBM) is a highly aggressive brain tumor with limited therapeutic options due to its invasive nature, therapeutic resistance, and the challenge of drug delivery across the blood–brain barrier (BBB). Mesenchymal stem cells (MSCs), owing to their tumor tropic properties and ability to cross the BBB, offer a promising platform for targeted anti-cancer delivery. We previously engineered MSCs to express CD::UPRT and TRAIL, along with chemokine receptors to enhance tumor homing (MSC-CD-TRAIL; BM03). This study evaluated the anti-tumor efficacy of BM03 using GBM patient-derived organoids (GBOs), clinically relevant in vitro models. Using a GBO–MSC co-culture system, BM03 significantly increased cell death and reduced viability in GBOs from four GBM patients compared with controls and MSC-WT groups. In 3D invasion assays, BM03-treated GBOs showed markedly reduced invasive outgrowth, accompanied by downregulation of EMT markers (Zeb1 and Snail) and stem-like markers (Olig2 and Sox2), particularly in invasive regions. GFAP expression remained unchanged, suggesting selective targeting of tumor stem-like cells. Live-cell imaging further demonstrated BM03 infiltration into GBOs, which was associated with increased apoptosis, as evidenced by elevated cleaved caspase-3 levels. These findings provide organoid-based preclinical evidence supporting further evaluation of BM03 as an MSC-based therapeutic strategy for GBM.

## 1. Introduction

Glioblastoma (GBM) is classified by the World Health Organization (WHO) as a grade 4 central nervous system (CNS) tumor, characterized by a poor prognosis and median survival of 12–15 months [1,2,3]. Surgical resection is still the primary treatment for GBM, typically followed by radiotherapy and temozolomide (TMZ)-based chemotherapy [4]. However, these standard therapies provide only modest survival benefits, extending median overall survival by approximately 2 months, while the 1-year survival rate remains low [5]. Current treatment challenges for GBM include therapeutic resistance, diffuse tumor infiltration, and the inability of most chemotherapeutic agents to penetrate the blood–brain barrier (BBB). Therefore, more effective treatments or drug delivery/targeting methods that can overcome these limitations and improve clinical outcomes are critically important.

Mesenchymal stem cells (MSCs) have emerged as promising therapeutic vehicles for cancer treatment due to their intrinsic tumor tropic properties and ability to survive within the brain microenvironment [6,7,8]. These characteristics make MSCs particularly attractive for the targeted delivery of anti-cancer agents in GBM [8,9,10]. MSCs have been engineered to deliver pro-apoptotic proteins, including tumor necrosis factor-related apoptosis-inducing ligand (TRAIL), which has demonstrated anticancer efficacy across multiple tumor types [11,12,13]. In addition, the suicide gene cytosine deaminase::uracil phosphoribosyltransferase (CD::UPRT) enhances therapeutic safety and efficacy by enabling localized conversion of the BBB-permeable prodrug 5-fluorocytosine (5-FC) into the cytotoxic agent 5-fluorouracil (5-FU), thereby inducing tumor-selective cell death [14,15]. This system is therefore particularly advantageous for treatment of brain tumors. Furthermore, genetic modification of MSCs to enhance tumor tropism, such as overexpression of chemokine receptors, has been actively investigated to improve targeted delivery efficiency [16,17].

Building on these strategies, we previously developed a multifunction-ally engineered MSC platform expressing TRAIL and CD::UPRT, along with chemokine receptors CCR2 and CXCR4 (MSC-CD-TRAIL; hereafter referred to as BM03). In our previous study, BM03 showed greater anti-tumor activity than single-module MSC-CD or MSC-TRAIL and demonstrated potent efficacy in both GBM cell lines and orthotopic xenograft mouse models [9]. However, these experimental models do not fully recapitulate the cellular heterogeneity and microenvironmental complexity of human GBM. In particular, two-dimensional cell cultures lack tumor complexity, while xenograft models do not adequately preserve patient-specific tumor characteristics or reliably predict therapeutic responses [18,19]. Patient-derived brain tumor organoids have recently emerged as advanced in vitro models that more faithfully recapitulate the histopathological architecture and cellular diversity of primary tumors [20,21,22]. Importantly, our well-characterized organoid system preserves key features such as patient-specific genetic alterations, histological characteristics, and tumor microenvironment [23,24], making it a relevant platform for preclinical therapeutic evaluation.

In this study, we evaluated the therapeutic efficacy of BM03 in patient-derived glioblastoma organoids (GBOs). We hypothesized that the multifunctional properties of BM03 would enable effective tumor targeting and enhanced therapeutic responses in a model that more closely recapitulates human GBM. Our findings extend the preclinical validation of BM03 to GBOs and demonstrate that its anti-tumor activity is maintained in a model that better preserves tumor heterogeneity, further supporting BM03 as a promising MSC-based therapeutic strategy for GBM.

## 2. Materials and Methods

### 2.1. Generation of GBOs from Patient Tissue and Maintenance

All tumor specimens were pathologically classified by a board-certified neuropathologist in accordance with the 2021 WHO Classification of Tumors of the Central Nervous System, 5th edition. Fresh GBM tissues were collected intraoperatively at the Neuro-oncology Center of Seoul St. Mary’s Hospital and immediately transferred into ice cold sterile phosphate-buffered saline (PBS). The derivation and culture of GBOs were performed according to previously established protocols used for our meningioma and gliosarcoma organoid models [23,24]. Briefly, resected tumor tissue was mechanically minced into fragments smaller than 1 mm^3^ without enzymatic digestion at 4 °C in Hibernate A medium (BrainBits LLC, Springfield, IL, USA) with appropriate additives. Organoids were stably established within 2 weeks and subsequently used for downstream assays. Detailed information about the patients is provided in Table 1. All procedures involving human tissue were conducted in compliance with the Declaration of Helsinki and approved by the Institutional Review Board of Seoul St. Mary’s Hospital (IRB approval number: KC21TISI0793; approval date: 30 December 2021). Written informed consent was obtained from all participants prior to sample collection.

### 2.2. Culture of MSC-WT and BM03

BM03 cells were previously established and extensively characterized [9]. Briefly, BM03 cells are genetically engineered MSCs that co-express CD::UPRT and TRAIL for GBM targeting, c-myc, hTERT, and tTA for immortalization, and CCR2 and CXCR4 to enhance tumor tropism, all introduced via lentiviral transduction. Both MSC-WT and BM03 cells were maintained in low-glucose Dulbecco’s Modified Eagle Medium (DMEM; WELGENE, Daegu, Republic of Korea) supplemented with 10% fetal bovine serum (FBS; Hyclone, Logan, UT, USA) and 1% penicillin–streptomycin (Thermo Fisher Scientific, Waltham, MA, USA) at 37 °C in a humidified atmosphere containing 5% CO_2_.

### 2.3. Co-Culture Conditions Between GBOs and MSCs

For the optimization of MSC seeding density, GBOs were co-cultured with 1 × 10^4^, 2 × 10^4^, or 5 × 10^4^ MSC-WT or BM03 cells per GBO, and viability was evaluated by WST assay after 72 h. Based on these results, a rate of 1 × 10^4^ cells per GBO was used for subsequent experiments. Individual GBOs were gently transferred using a wide-bore pipette tip to minimize mechanical disruption, and repeated pipetting was avoided during handling. Each GBO was placed in an individual well of a 24-well plate, and 1 × 10^4^ MSC-WT or BM03 cells were seeded into each well containing a single organoid. Co-cultures were incubated at 37 °C in a humidified 5% CO_2_ atmosphere without agitation for 6 h to allow cellular attachment and stabilization. Following this incubation period, 5-FC (100 µg/mL) was added to the BM03 group, and the plates were transferred to an orbital shaker operating at 120 rpm, consistent with our previously established patient-derived brain tumor organoid culture protocols [23,24]. The co-cultures were maintained under these conditions for the remaining 66 h, resulting in a total co-culture period of 72 h. The same shaking conditions were applied to all experimental groups.

### 2.4. Organoid Viability and Cytotoxicity Test

The viability of individual GBOs was assessed by WST analysis using the Cell Counting Kit-8 (CCK-8) reagent (Dojindo Laboratories, Kumamoto, Japan). Each GBO was initially measured at 0 h before co-culture and subsequently followed under the same experimental conditions. For each measurement, GBOs were gently washed with PBS and transferred to individual wells of a 96-well plate containing 100 μL of fresh medium. A 10% dilution of CCK-8 reagent was added to each well, and the plate was incubated for 1.5 h. Absorbance was measured using a microplate reader (SYNERGY H1, BioTek, Winooski, VT, USA) at 450 nm. Viability at 72 h was normalized to the corresponding 0 h value of the same organoid to minimize inter-organoid variability associated with differences in organoid size, architecture, and reagent penetration.

### 2.5. Live/Dead Fluorescence Staining

GBOs co-cultured with MSC-WT or BM03 for 72 h were transferred to individual wells of a 96-well plate and washed twice with PBS to minimize the contribution of MSCs remaining outside or attached to the GBOs. Organoids were then washed once more with fresh GBO culture medium on a shaker for 30 min. For live/dead staining, Calcein-AM and propidium iodide (PI) from the Live/Dead Cell Staining Kit (Dynebio, Seongnam, Republic of Korea) were diluted in PBS to final concentrations of 1 μM and 5 μM, respectively, and 100 μL of the staining solution was added to each well. Plates were incubated at 37 °C for 20 min, and fluorescence signals were examined within the GBO region. Live cells were visualized by Calcein-AM fluorescence using a 490 nm excitation filter, whereas dead cells were selectively visualized by PI fluorescence using a 545 nm excitation filter.

### 2.6. Three-Dimensional (3D) Invasion Assay

To assess the impact of BM03 on cancer invasiveness, a 3D invasion assay was performed on GBO. Each organoid was embedded in a matrix containing a mixture of type I collagen (Nita Gelatin, Osaka, Japan) and Matrigel (Corning, Corning, NY, USA). Collagen type 1 and Matrigel were mixed in 2x Ham’s F12 medium at a 1:1 ratio on ice, and 10% reconstitution buffer (0.002 g/mL NaHCO3, 0.0047 g/mL HEPES, and 0.005 N NaOH) was added to adjust pH. The matrix solution (100 µL) was transferred into each well of a 96-well plate, and a single organoid was embedded into the matrix solution before gelation. The matrix was solidified for 30 min, and culture medium was added on top of the gelled matrix. The relative invasion area of each GBO was quantified by normalizing to 0 d.

### 2.7. Histology, Immunofluorescence (IF), and Whole-Mount Fluorescence Staining

GBOs were fixed in 4% paraformaldehyde (Biosesang, Yongin, Republic of Korea) for 1 h at RT. For cryosectioning, fixed GBOs were immersed in 30% sucrose until fully submerged. For paraffin embedding, fixed GBOs were first embedded in 2% agarose, followed by standard dehydration and paraffin infiltration protocols. Paraffin-embedded GBOs were sectioned at a thickness of 4 μm, while cryosections were prepared at 8–10 µm thickness. Hematoxylin–eosin (H&E) staining was performed according to the manufacturer’s instructions and scanned using Pannoramic SCAN II (3DHISTECH Ltd., Budapest, Hungary). Representative images were captured using CaseViewer version 2.4 and Automated Slide Analysis Platform.

For immunofluorescence (IF) analysis, tissue sections were washed with PBS containing 0.1% Tween-20 (PBST). Antigen retrieval was conducted by microwave heating in citrate buffer for 15 min to facilitate heat-induced epitope retrieval. Permeabilization was performed using a buffer containing 1% bovine serum albumin (BSA; BioWorld, Dublin, OH, USA) and 0.25% Triton X-100 (AMRESCO, Solon, OH, USA) in PBS, followed by blocking with 5% normal goat serum or BSA. Slides were incubated overnight at 4 °C with primary antibodies, followed by 1 h incubation at RT with appropriate secondary antibodies. Details of all antibodies used are provided in Table S1. Nuclei were counterstained with Hoechst for 10 min. Fluorescent images were acquired under a confocal laser scanning microscope (LSM800 with Airyscan, Carl Zeiss Microscopy GmbH, Jena, Germany).

For whole-mount immunofluorescence analysis, GBOs were pre-stained with Hoechst, while MSC-WT and BM03 cells were separately labeled with 10 μM CellTracker™ Green CMFDA dye (Invitrogen, Paisley, Scotland, UK) for 30 min in a humidified 37 °C incubator with 5% CO_2_. Hoechst-labeled GBOs were then co-cultured with CMFDA-labeled MSC-WT or BM03 cells for the indicated time periods. Following co-culture, GBOs were washed with PBS to remove MSCs remaining outside or loosely attached to the organoids and fixed. Fixed intact GBOs were blocked and permeabilized in PBS containing 2% normal goat serum, 1% BSA, 0.5% Triton X-100, and 0.5% Tween-20 for at least 6 h at 4 °C. GBOs were then incubated overnight at 4 °C with a cleaved caspase-3 antibody, followed by an anti-rabbit IgG-CFL 594 secondary antibody. After washing, GBOs were cleared overnight at RT using RapiClear 1.47 (SunJin Lab Co., Hsinchu City, Taiwan) and imaged using a confocal laser scanning microscope (FV3000, Evident, Tokyo, Japan). Z-stack images were acquired throughout the organoid depth and processed using Z-intensity projection. For quantitative image analysis, the total GBO area was defined as a region of interest (ROI) based on the Hoechst-stained organoid boundary. Within each GBO ROI, CMFDA-positive and cleaved caspase-3-positive areas were quantified using ImageJ version 1.54t. Fluorescence-positive areas were identified by threshold-based segmentation using consistent analysis settings across images and were expressed as percentages of the total GBO ROI area.

### 2.8. Western Blotting

GBOs were transferred to 1.5 mL microcentrifuge tubes and lysed in RIPA buffer supplemented with protease and phosphatase inhibitors (GenDEPOT, Katy, TX, USA) for 1 h on ice. Lysates were centrifuged at 13,000 rpm for 20 min at 4 °C, and the resulting supernatants were collected and stored at −80 °C until use. Protein quantification was performed using Bio-Rad protein colorimetric assay dye (Bio-Rad Laboratories Inc., Hercules, CA, USA). Twenty μg of total protein per sample was separated by SDS-PAGE and transferred to a PVDF membrane, which was blocked with 5% BSA for 1 h. Subsequently, the membrane underwent overnight incubation with specific primary antibodies. The membrane was washed with TBS with 0.1% Tween-20 (TBST) and incubated with secondary antibody for 1 h at RT. Following additional washes with TBST, the membrane was developed using ECL solutions (SuperSignal™ West Pico PLUS Chemiluminescent Substrate and SuperSignal™ West Femto Maximum Sensitivity Substrate; Thermo Fisher Scientific) and imaged using a bioimage analyzer (Amersham Imager 600, Fuji Photo Film Co., Ltd., Tokyo, Japan). Densitometric quantification was performed using Image J software. A complete list of antibodies used for Western blotting is provided in Table S1.

### 2.9. Live Cell Imaging for Cell Tracking

To assess the infiltration of MSC-WT and BM03 into GBOs, both cell types were fluorescently labeled with CellTracker™ Green CMFDA dye (Invitrogen), as described above. Simultaneously, GBOs were stained with Hoechst dye (Thermo Fisher Scientific) for 10 min at RT, followed by thorough washing with PBS to remove excess dye. Fluorescently labeled MSC-WT and BM03 were then independently seeded onto the Hoechst-stained GBOs. Real-time tracking of cell infiltration was performed using the Lionheart FX Automated Microscope (BioTek Instruments, Inc., Winooski, VT, USA). Images were acquired at 1 h intervals to generate time-lapse sequences, enabling the visualization and analysis of the dynamic infiltration behavior of the labeled cells into the GBOs.

### 2.10. Statistical Analysis and Software

Statistical analysis was performed using GraphPad Prism version 9.0.0 (GraphPad Software, San Diego, CA, USA). Specific statistical methods applied to each dataset are detailed in the corresponding figure legends. Comparisons among multiple treatment groups were performed using one-way ANOVA, followed by Tukey’s multiple-comparisons test. Repeated-measures ANOVA was used for longitudinal measurements, as specified in the corresponding figure legends. For analyses involving two factors with repeated measurements, two-way mixed ANOVA followed by Sidak’s multiple-comparisons test was used. Data are presented as mean ± SEM.

## 3. Results

### 3.1. BM03 Induces Cytotoxicity in GBOs

**Figure 1:**
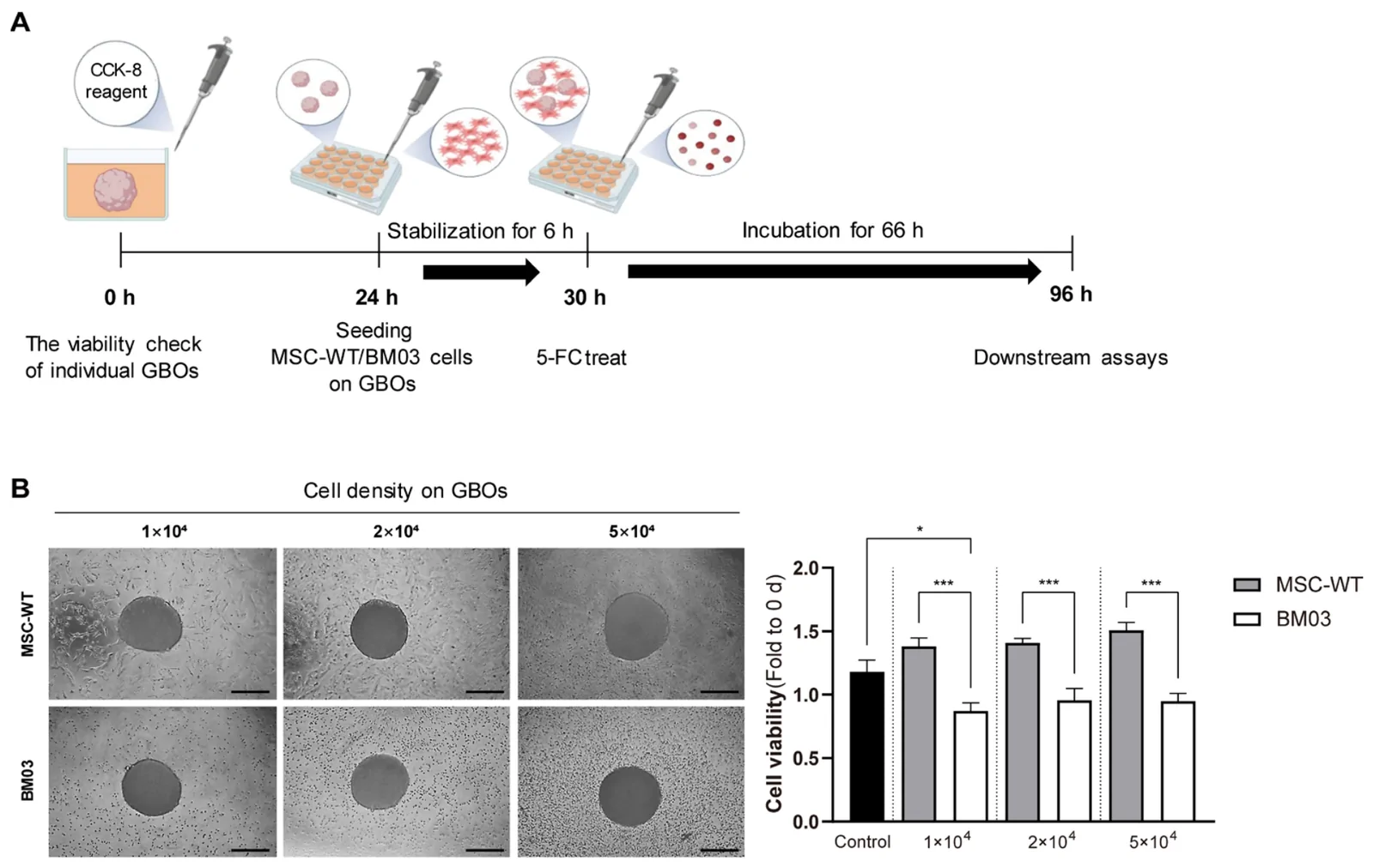
Co-culture condition of MSCs and GBOs. (**A**) The diagram illustrates the overall experimental design used to evaluate the therapeutic effects of MSC-WT and BM03 in GBOs. Initially, GBO viability was assessed using the WST assay to evaluate baseline viability prior to co-culture. GBOs were then co-cultured with either MSC-WT or BM03. After 6 h, 5-FC was added to the BM03 group to initiate prodrug conversion. Following 72 h of incubation, GBOs co-cultured with MSC-WT or BM03 underwent downstream analyses. (**B**) Bright-field images of GBOs (GBO21-07) co-cultured with MSC-WT or BM03 at different cell densities (1 × 10^4^, 2 × 10^4^, and 5 × 10^4^). Images were acquired at 4× magnification. Scale bar = 500 µm. (**C**) Quantification of GBO21-07 viability after 72 h of co-culture under each condition using the WST assay. Data were obtained from three independent experiments (*n* = 10 per group). Viability at 72 h was normalized to the corresponding baseline value at 0 h for each individual GBO and expressed as fold change. Statistical significance (one-way ANOVA with Tukey’s post hoc test) was determined, and the results were reported as the means ± SEM (* *p* < 0.05 and *** *p* < 0.001).

**Figure 2:**
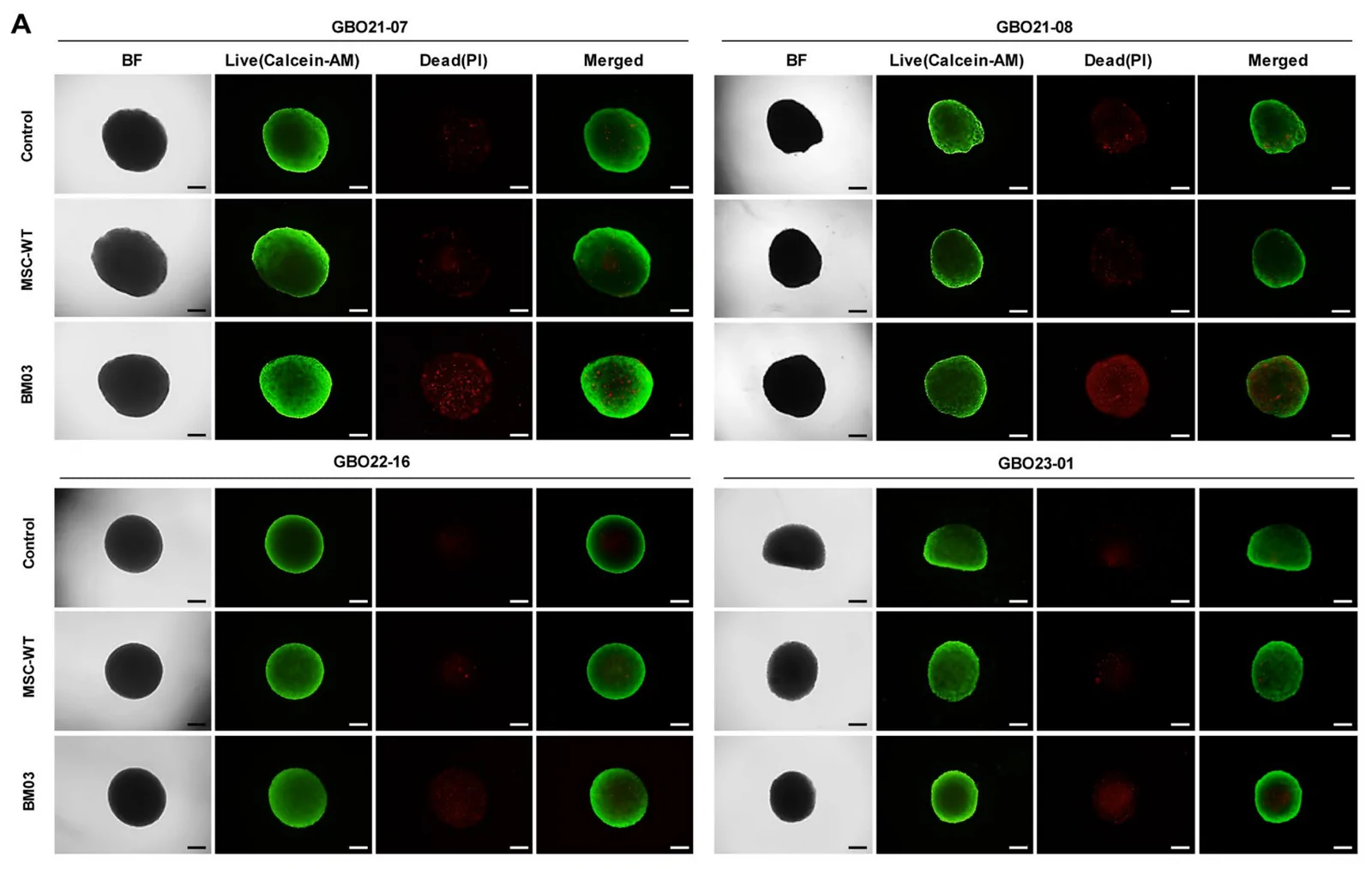

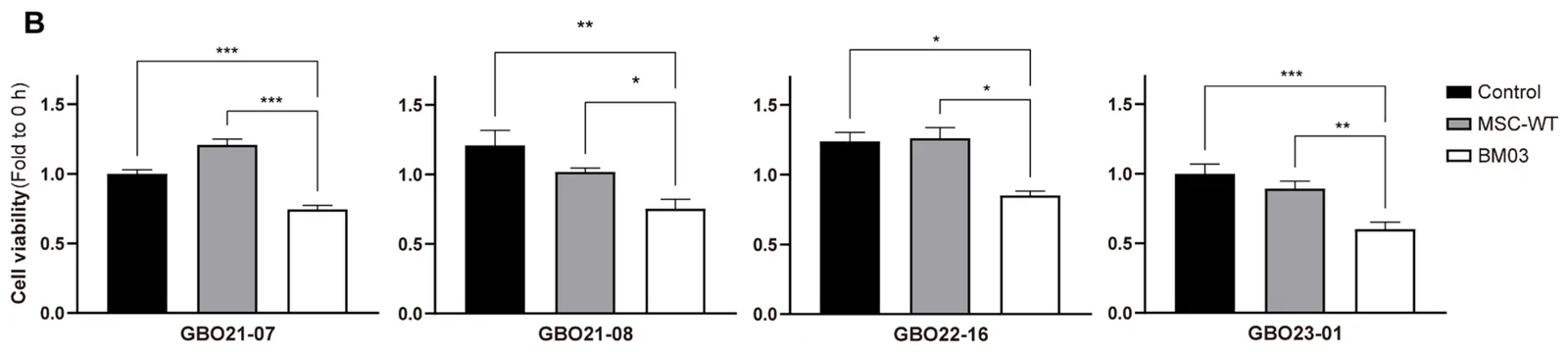
Cell viability of GBOs after MSC-WT and BM03 cell treatment. (**A**) live/dead cell staining of GBOs after co-culturing MSC-WT and BM03. At least two GBOs per group were analyzed in each independent experiment. Live cells were labeled with calcein-AM (green fluorescence), and dead cells were stained with propidium iodide (PI, red fluorescence). Scale bar = 500 µm (magnification 6×). (**B**) Viability of GBOs derived from four patients was evaluated by WST assay after 72 h. The numbers of GBOs analyzed per group were *n* = 13 for GBO21-07, *n* = 4 for GBO21-08, *n* = 5 for GBO22-16, and *n* = 11 for GBO23-01. Viability at 72 h was normalized to the corresponding baseline value at 0 h for each individual GBO and expressed as fold change. Statistical significance (one-way ANOVA with Tukey’s post hoc test) was determined, and the results were reported as the means ± SEM (* *p* < 0.05, ** *p* < 0.01, and *** *p* < 0.001).

To establish an effective co-culture system for evaluating the cytotoxic effects of MSC-WT and BM03 on GBOs, we first optimized the seeding density of both MSCs. The experimental timeline, from cell seeding to downstream assays, is illustrated (Figure 1A). Three seeding densities, namely −1 × 10^4^, 2 × 10^4^, and 5 × 10^4^ MSC-WT or BM03 per GBO, were tested (Figure 1B,C). Among these, the 1 × 10^4^ seeding density provided optimal conditions for comparing cytotoxicity and was therefore selected for subsequent experiments. We next investigated the cytotoxic effects of BM03 against GBOs from four patients diagnosed with GBM. Clinical and molecular characteristics of the corresponding patient tumors are summarized in Table 1. The four GBO models were further characterized for histological, glial phenotypic, growth/viability, and invasive features (Figure S1). Live/dead fluorescence staining revealed an increased proportion of dead cells, indicated by PI staining, in the BM03-treated group compared to both untreated controls and MSC-WT groups (Figure 2A). To further confirm these results, we performed cell viability assays. GBOs co-cultured with BM03 showed a significant decrease in cell viability relative to the control and MSC-WT groups (Figure 2B). The cytotoxic effect of BM03 was further enhanced by CD::UPRT/5-FC-dependent prodrug conversion, as demonstrated by the greater reduction in GBO viability following BM03 + 5-FC treatment compared with BM03 alone (Figure S2). Collectively, these findings demonstrate that BM03 exerts a pronounced cytotoxic effect on GBOs.

### 3.2. BM03 Attenuates Invasive Behavior and Stemness in GBOs

**Figure 3:**
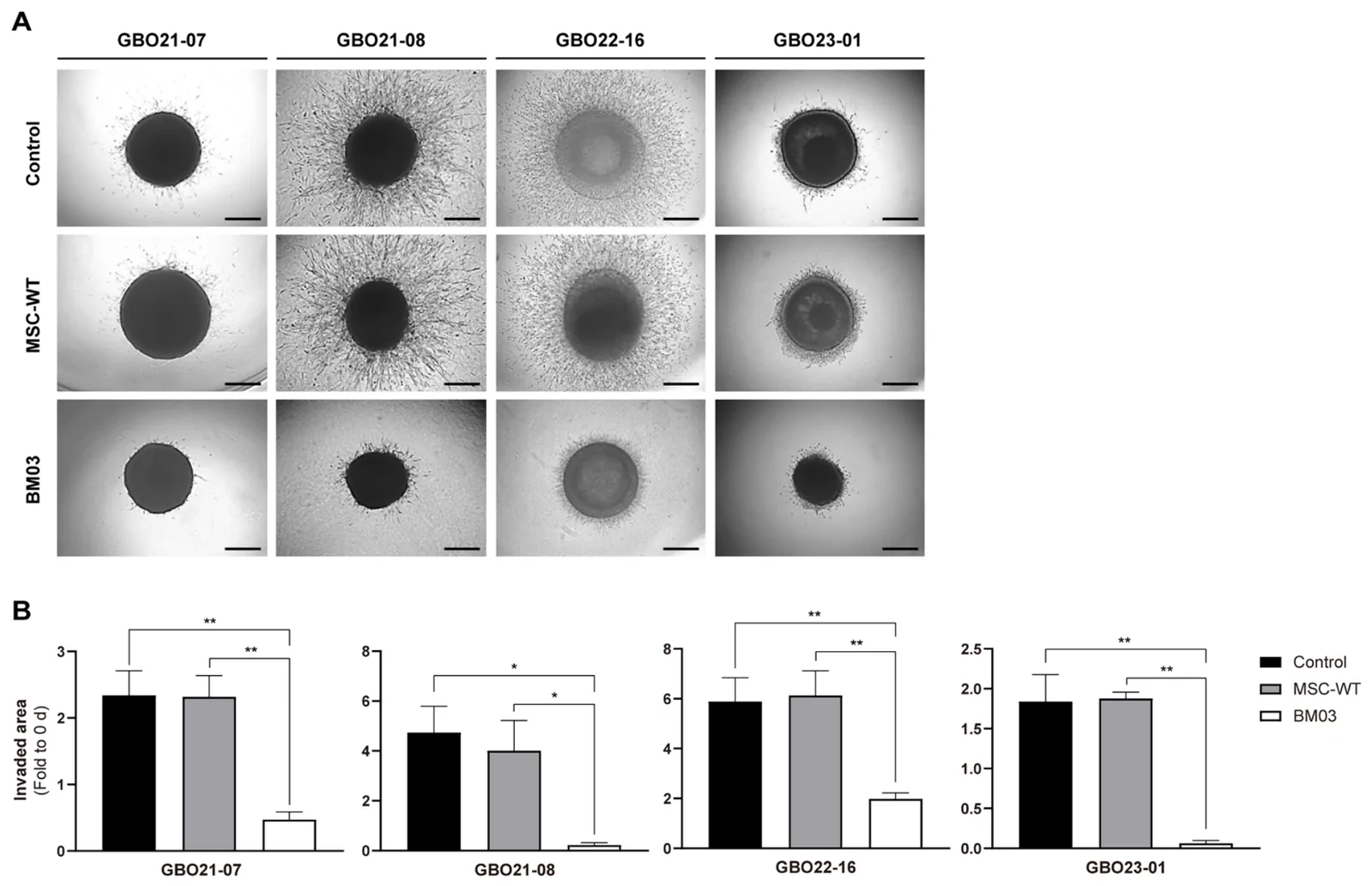

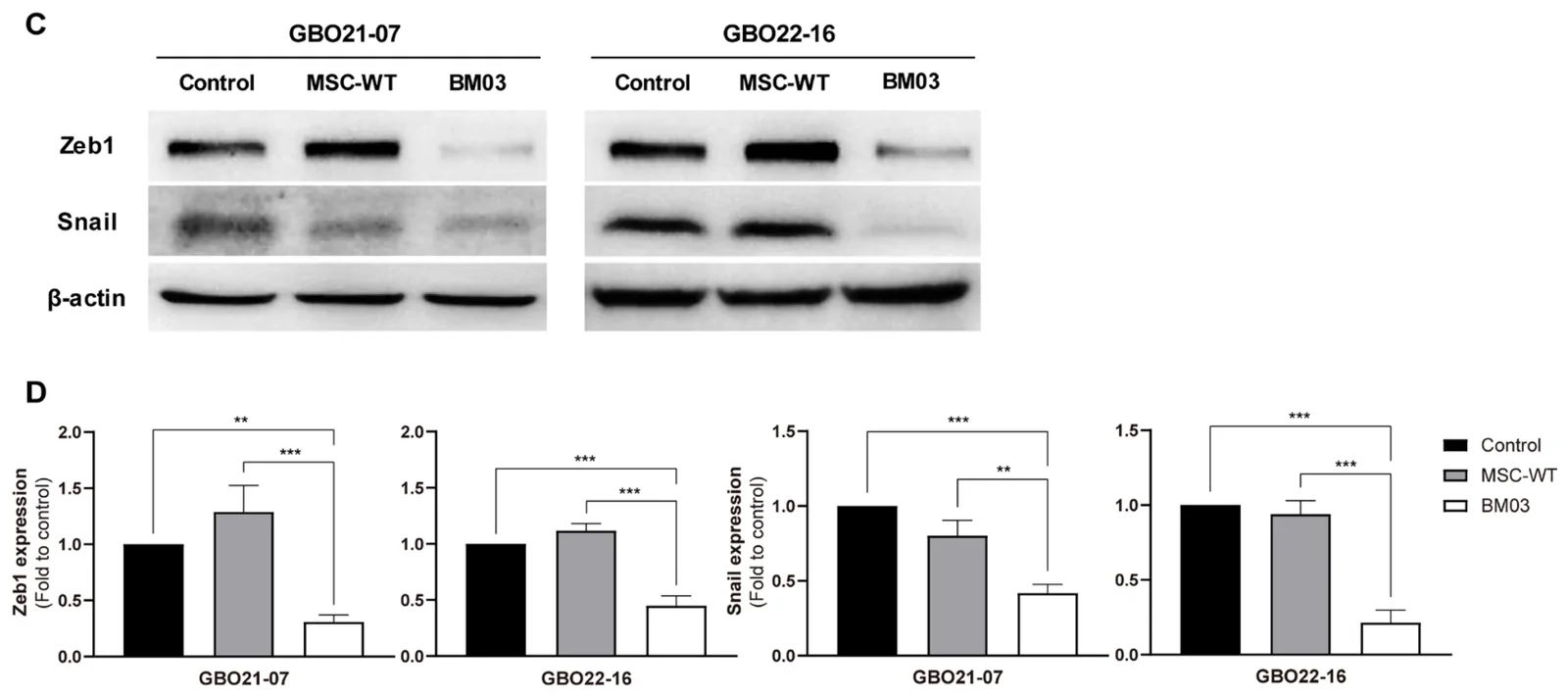
BM03 inhibits invasive behaviors of GBOs. (**A**) Three-dimensional (3D) organoid invasion models were established by embedding the organoids in a gel composed of Matrigel and collagen. GBOs were co-cultured with MSC-WT or BM03 for 72 h. After the initial 6 h of co-culture, 5-FC was added to the BM03 group. The GBOs were then embedded in the gel matrix. Bright-field images were acquired at 0 d and 7 d post-embedding to assess organoid invasion. Scale bar = 500 µm (magnification 5×). (**B**) Quantification of the invaded area of GBOs treated with MSC-WT or BM03. The numbers of GBOs analyzed per group were *n* = 6 for GBO21-07, *n* = 4 for GBO21-08, *n* = 8 for GBO22-16, and *n* = 3 for GBO23-01. The invaded area at 7 d was normalized to the corresponding area at 0 d for each individual GBO and expressed as fold change. Statistical significance (one-way ANOVA with Tukey’s post hoc test) was determined, and the results were reported as the means ± SEM (* *p* < 0.05 and ** *p* < 0.01). (**C**) Representative images of Western blot show the expression of Zeb1 and Snail in GBOs (GBO21-07 and 22-16) treated with MSC-WT and BM03. β-actin was used as a loading control. All Western blot experiments were performed in three independent experiments. (**D**) Quantification of Zeb1 and Snail protein expression in GBOs treated with MSC-WT or BM03. Densitometric values were normalized to β-actin and expressed as fold changes relative to the untreated control group. Statistical significance (one-way ANOVA with Tukey’s post hoc test) was analyzed, and the results were reported as the means ± SEM (** *p* < 0.01 and *** *p* < 0.001).

To evaluate the impact of BM03 on the invasiveness of GBOs, a 3D invasion assay was performed using collagen and Matrigel. After 7 days, both the untreated and MSC-WT-treated GBOs demonstrated a significant increase in invasive behavior. In contrast, GBOs treated with BM03 exhibited either no invasion or markedly reduced invasive behavior (Figure 3A). These observations were corroborated by quantification of the invaded area (Figure 3B). To further investigate the expression levels of proteins associated with invasion, Western blot analysis was performed. Treatment with BM03 resulted in a substantial reduction in the expression of Zeb1 and Snail, key transcription factors implicated in epithelial–mesenchymal transition (EMT) and tumor invasiveness (Figure 3C,D and Figure S3).

**Figure 4:**
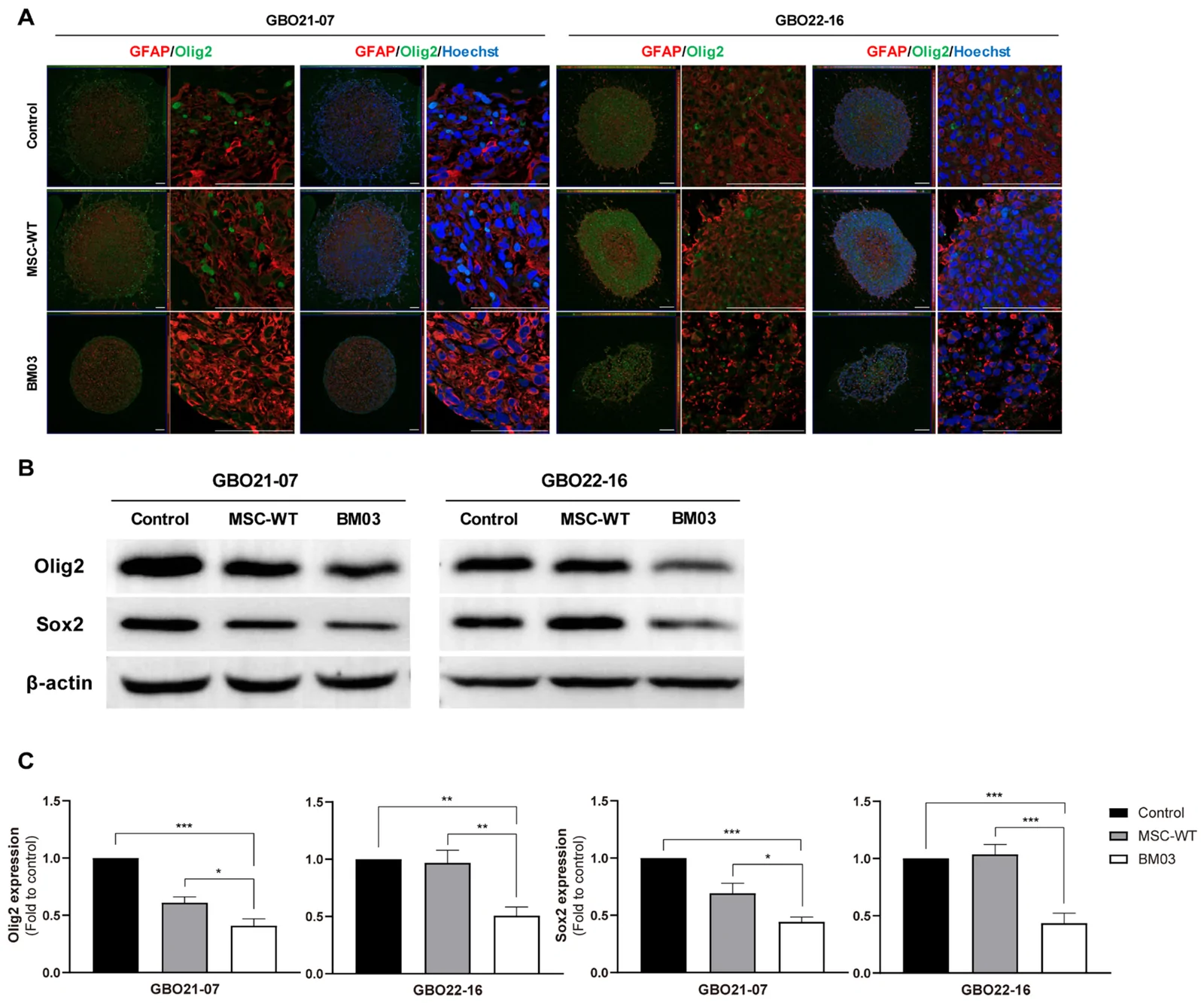
BM03 attenuates stemness in GBOs. (**A**) Representative immunofluorescence for Olig2 (green) and GFAP (red), and Hoechst (blue) in GBOs (GBO21-07 and 22-16) treated with MSC-WT and BM03. At least three GBO sections per group were analyzed. Scale bar = 100 μm. (**B**) Representative images of Western blot show the protein expression of Olig2 and Sox2 in GBOs co-cultured with MSC-WT and BM03 for 72 h. (**C**) Densitometric values were normalized to β-actin and then normalized to the untreated control group, with results expressed as fold changes relative to the control. Data were obtained from three independent experiments. Statistical significance (one-way ANOVA with Tukey’s post hoc test) was determined, and the results were reported as the means ± SEM (* *p* < 0.05, ** *p* < 0.01, and *** *p* < 0.001).

Subsequently, to examine the effects of BM03 on cancer stemness within GBOs, IF and Western blot analyses were performed. IF results indicated no significant change in the expression of GFAP, an astrocyte marker, across all treatment groups. However, nuclear expression of Olig2 was enriched at the periphery of GBOs in both untreated and MSC-WT treated groups, whereas a notable reduction in Olig2 expression was observed in the BM03-treated GBOs (Figure 4A). These findings were confirmed by Western blot (Figure 4B,C and Figure S4). Furthermore, the protein expression levels of Olig2 and Sox2, both recognized markers of glioblastoma stem-like cells, were significantly decreased in GBOs treated with BM03 compared to the untreated- and MSC-WT treated groups. Collectively, these results suggest that BM03 treatment attenuates both the invasive properties and stem-like characteristics of GBOs.

### 3.3. BM03 Exhibits Enhanced Tumor Tropism and Induces Apoptosis in GBOs

To evaluate the enhanced tumor tropism of BM03, we utilized fluorescent labeling to monitor its migratory behavior toward GBOs. GBOs were stained with Hoechst dye, while MSC-WT or BM03 were transiently labeled with CMFDA. Time-lapse imaging demonstrated that BM03 exhibited greater migration toward GBOs, with apparent accumulation toward the GBO core (Movie S1A,C), whereas MSC-WT remained predominantly at the GBO periphery and showed relatively limited inward migration at the same time points (Movie S1B,D).

**Figure 5:**
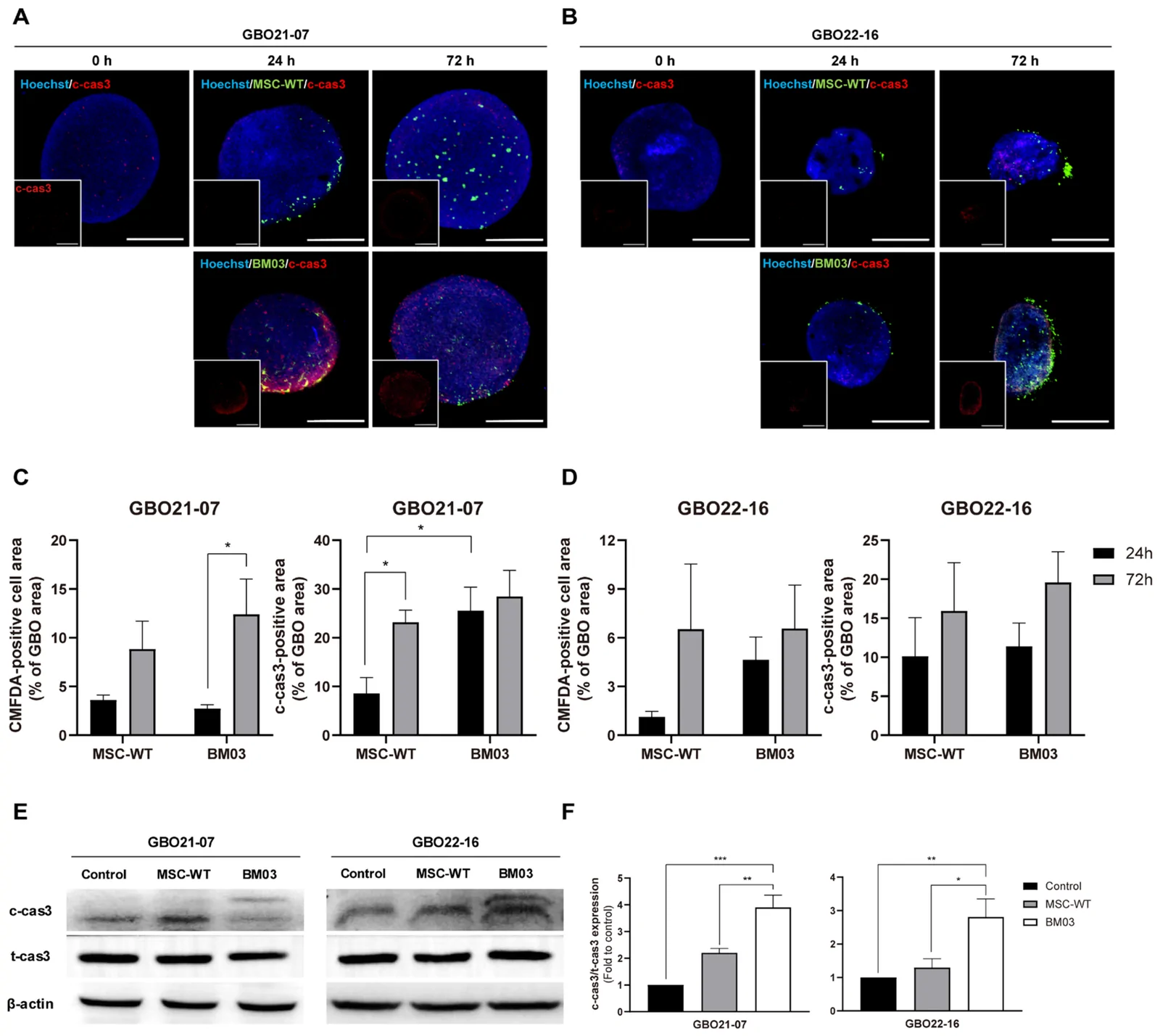
BM03 exhibits enhanced penetration into GBOs accompanied by increased apoptosis. (**A**,**B**) Representative whole-mount fluorescence images of (**A**) GBO21-07 and (**B**) GBO22-16 co-cultured with MSC-WT or BM03. GBOs were stained with Hoechst (blue), MSC-WT and BM03 cells were labeled with CMFDA (green), and apoptosis was assessed by cleaved caspase-3 (c-cas3; red) staining. Images were acquired at 24 and 72 h using confocal microscopy with 3D z-stack imaging. Insets show the cleaved caspase-3 channel alone. Scale bars, 500 μm; inset scale bars, 500 μm. (**C**,**D**) Quantification of the CMFDA-positive MSC area and c-cas3-positive area in (**C**) GBO21-07 and (**D**) GBO22-16 at 24 and 72 h. The total GBO area was defined as the region of interest (ROI), and the CMFDA-positive and c-cas3-positive areas within the ROI were quantified and expressed as percentages of the total GBO area. GBO21-07 included MSC-WT (*n* = 4) and BM03 (*n* = 3), whereas GBO22-16 included MSC-WT (*n* = 3) and BM03 (*n* = 3). Statistical significance in (**C**,**D**) was assessed using two-way ANOVA followed by Šídák’s multiple-comparisons test (* *p* < 0.05). (**E**) Representative Western blot analysis of apoptosis-related proteins, including c-cas3 and total caspase-3 (t-cas3) in GBO21-07 and GBO22-16 following co-culture with MSC-WT or BM03. β-actin was used as a loading control. (**F**) Densitometric quantification of the Western blot results shown in (**E**). Protein expression levels were normalized to β-actin and expressed as fold changes relative to the control group. Data was obtained from three independent experiments (*n* = 3) and are presented as mean ± SEM. Statistical significance was assessed using one-way ANOVA followed by Tukey’s multiple-comparisons test (* *p* < 0.05, ** *p* < 0.01, and *** *p* < 0.001).

To further evaluate BM03 penetration within GBO structure, we performed whole-mount fluorescence imaging with 3D z-stack analysis to directly evaluate the infiltration of CMFDA-labeled MSCs and its association with apoptosis. Whole-mount imaging revealed greater accumulation of CMFDA-labeled BM03 within the GBO, including the core region, whereas MSC-WT showed relatively limited internal penetration (Figure 5A,B). Increased BM03 accumulation within the GBO was associated with enhanced cleaved caspase-3 expression (Figure 5A,B), and quantitative analysis further confirmed increased CMFDA fluorescence within the GBO core in the BM03-treated group (Figure 5C,D). Western blot analysis further supported increased cleaved caspase-3 expression following BM03 treatment (Figure 5E,F and Figure S5). Together, these findings demonstrate enhanced migration and penetration of BM03 into GBOs and show that BM03 accumulation is associated with increased apoptosis.

## 4. Discussion

**Figure 6:**
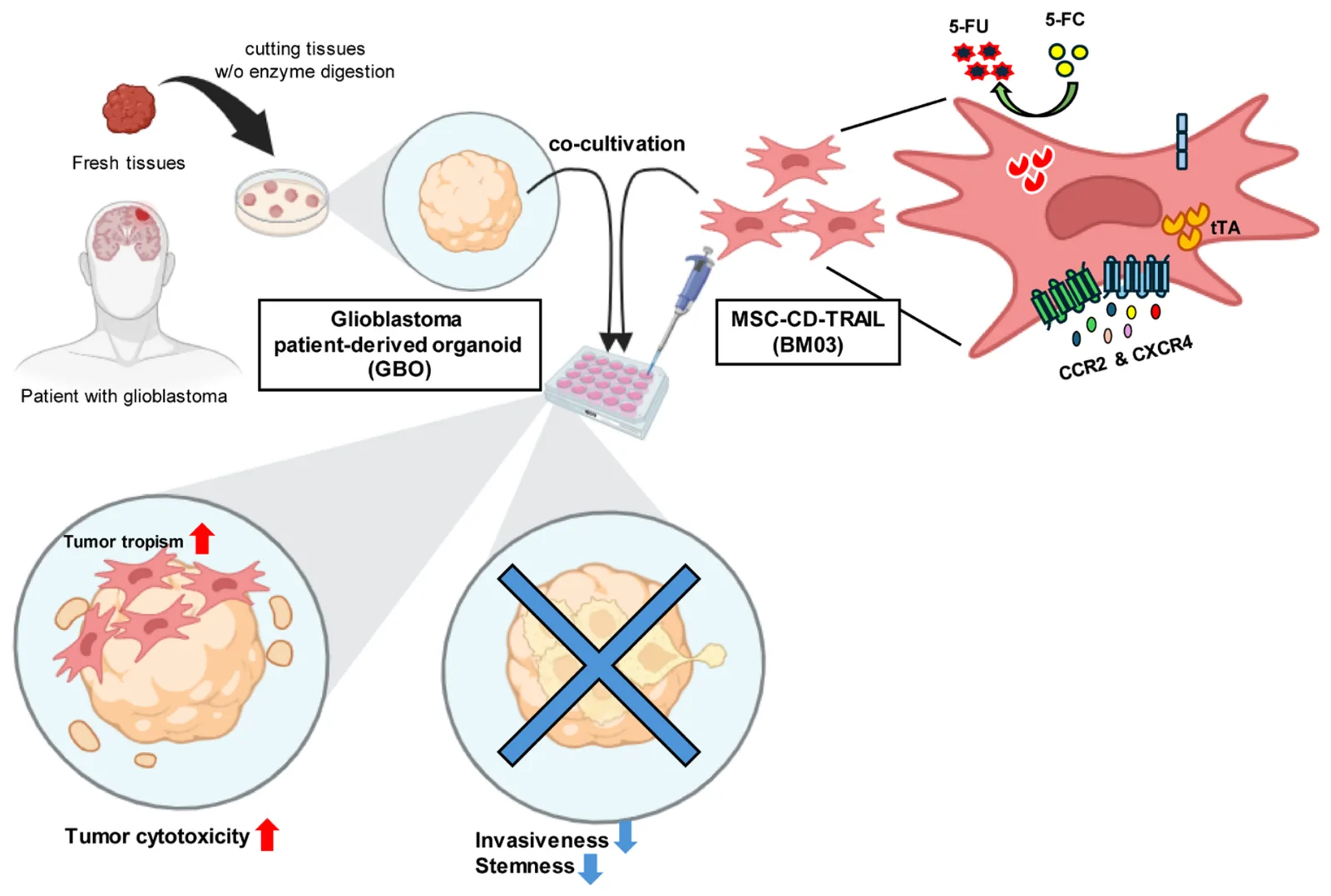
Schematic overview of the study design and findings. Patient-derived glioblastoma organoids (GBOs) and engineered mesenchymal stem cells expressing CD::UPRT and TRAIL (MSC-CD-TRAIL; BM03) were generated. Co-culture of BM03 with GBOs was used to evaluate anti-tumor effects. BM03 showed enhanced tumor tropism compared to MSC-WT and induced significant cytotoxicity in GBOs. Additionally, BM03 treatment reduced GBO invasiveness and stemness. These findings suggest that BM03 is a promising therapeutic approach for refractory GBM.

Here, we evaluated a multifunctional, genetically engineered MSC platform co-expressing TRAIL and CD::UPRT (MSC-CD-TRAIL; BM03) using GBM patient-derived organoids (GBOs). Using this patient-derived model that preserves key molecular and histopathological features of primary tumors, we demonstrate that BM03 exerts potent anti-tumor effects, including robust induction of apoptosis, suppression of cancer stem-like phenotypes, and inhibition of tumor invasion (Figure 6). Importantly, building on our previous findings in GBM cell lines and xenograft models, this study demonstrates that the efficacy of BM03 is not limited to simplified models but is robustly maintained in patient-derived organoids. These results provide strong preclinical validation of BM03 and highlight its potential as a next-generation cell-based therapeutic strategy for GBM.

A key advance of the present study is the functional validation of BM03-induced cytotoxicity in patient-derived organoids, which better capture tumor heterogeneity than conventional models. In this context, BM03 exhibits pronounced cytotoxicity in GBOs, which was not observed following MSC-WT treatment. Additional analysis comparing BM03 treatment in the presence or absence of 5-FC showed that BM03 + 5-FC reduced GBO viability more effectively than BM03 alone, whereas 5-FC alone or MSC-WT + 5-FC showed no substantial cytotoxicity (Figure S2). These findings indicate that CD::UPRT/5-FC-mediated prodrug conversion further enhances BM03 cytotoxicity, whereas the 5-FC-independent component, including TRAIL-associated activity, was relatively limited under the tested conditions. These findings are consistent with our previous study showing that the multifunctional BM03 platform exhibited greater anti-tumor activity than single-module MSC-CD or MSC-TRAIL in both in vitro and in vivo models [9] and further support the reproducibility of BM03-mediated anti-tumor activity in patient-derived organoids. Notably, the use of 5-FC—which efficiently crosses the BBB—further strengthens the translational potential of this platform for in vivo applications. The cytotoxic effects of BM03 likely reflect an amplified, rather than merely additive, apoptotic response, potentially facilitated by tumor-homing properties of the engineered MSCs and pro-apoptotic factors such as IFN-β, FasL, and exosomal miRNAs [7,25]. Together, these findings support the multimodal anti-tumor activity of BM03, in which CD::UPRT/5-FC-mediated cytotoxicity contributes alongside TRAIL-associated activity and the tumor-homing properties of the engineered MSCs. Nevertheless, the potential immunosuppressive role of MSCs warrants further investigation into their dualistic function in tumor biology. 

Beyond cytotoxicity, our findings demonstrate that BM03 significantly impairs the invasive potential of GBOs, an effect not captured in our previous models. Tumor invasion into surrounding brain tissue remains one of the most intractable features of GBM and a major contributor to recurrence [26,27]. The reduced invasive outgrowth from organoids in 3D matrix cultures following BM03 treatment, accompanied by downregulation of EMT markers Zeb1 and Snail, suggests disruption of transcriptional programs underpinning tumor dissemination. This dual activity—inducing apoptosis and repressing invasion—is especially compelling given the increasing recognition of mesenchymal transition as a key driver of both therapy resistance and immune evasion in GBM [28,29]. Furthermore, our findings show the downregulation of glioma stem-like cell markers Olig2 and Sox2—key transcription factors associated with tumor-initiating potential and therapeutic resistance [30,31,32]—in BM03-treated GBOs. The pronounced depletion of Olig2 in the peripheral, invasive zones of GBOs following BM03 exposure suggests that the engineered MSCs not only penetrate the organoid core but also impact the most therapeutically recalcitrant subpopulations. Importantly, these effects were achieved without altering astrocytic differentiation, as evidenced by stable GFAP expression. These data highlight a degree of lineage specificity and underscore the ability of BM03 to engage and disrupt core transcriptional programs that sustain GBM aggressiveness. Another salient feature of our platform is the markedly enhanced tumor tropism of BM03. Time-lapse imaging revealed accelerated and preferential migration of BM03 toward GBOs, while whole-mount 3D imaging further demonstrated greater penetration into the GBO core. These findings suggest that the enhanced expression of CCR2 and CXCR4 in engineered MSCs facilitates rapid and directed migration toward GBM-secreted chemokines (e.g., SDF-1α and MCP-1) [9,33,34], enabling deep tumor penetration. This chemotactic responsiveness is critical for efficient payload delivery within the dense tumor mass—a longstanding hurdle in GBM therapy. Importantly, the in vivo tumor-targeting and therapeutic efficacy of BM03 were previously demonstrated in an immunosuppressed orthotopic GBM xenograft model [9]. The present GBO findings therefore further support the cross-platform reproducibility of BM03 tumor tropism and anti-tumor activity in a patient-derived model. However, the current findings should be interpreted as organoid-based preclinical evidence rather than direct evidence of clinical applicability. Furthermore, evaluation of BM03 in an immunocompetent brain milieu remains necessary because neither the xenograft nor the GBO model fully recapitulates an intact immune microenvironment. Thus, further studies in advanced in vivo models, particularly immunocompetent GBM models, will be required to evaluate tumor targeting, immune interactions, biodistribution, safety, and therapeutic efficacy.

Several limitations of the present study should be acknowledged. The use of four patient-derived GBO models limits the generalizability of the findings across the substantial inter-patient heterogeneity of GBM, and larger, molecularly diverse GBO cohorts will be required to evaluate variability in therapeutic response. In addition, although GBOs preserve important tumor features, they do not fully recapitulate the immune, vascular, anatomical, and pharmacokinetic complexity of the in vivo brain tumor microenvironment. The present study also did not directly assess BM03 toxicity toward normal brain-derived cells or tissues. Future studies using normal brain-derived models and appropriate in vivo safety models will therefore be needed to evaluate potential off-target toxicity and tumor selectivity. In addition, combination strategies with immunomodulatory therapies or chemotherapies may warrant further investigation. In particular, the integration of this cell-based platform with immune checkpoint inhibitors or agents that reprogram the tumor microenvironment may offer additional benefits. Moreover, given the modular nature of the MSC delivery system, further optimization—such as incorporating tumor-targeting ligands or cytokine payloads—may enable precision targeting of diverse molecular GBM subtypes. Overall, the present study broadens the preclinical evaluation of BM03 by demonstrating its multifaceted anti-tumor activity in GBOs that better preserve tumor heterogeneity.

## 5. Conclusions

In this study, BM03, a multifunctionally engineered MSC platform expressing CD::UPRT and TRAIL, demonstrated significant anti-tumor activity in GBOs. BM03 reduced GBO viability, induced apoptosis, suppressed invasive behavior, and decreased EMT- and stemness-associated markers. In addition, its enhanced migration and infiltration into GBOs support its potential as a targeted cell-based delivery platform for GBM. These findings extend the preclinical validation of BM03 to patient-derived organoid models that better preserve tumor heterogeneity. Together with our previous in vivo findings, the present study provides complementary preclinical evidence supporting BM03 as a mechanistically rational MSC-based therapeutic strategy for GBM. Its tumor-homing properties and multimodal cytotoxic activity, now further validated in patient-derived organoids, provide a strengthened preclinical basis for future translational development.

## Figures and Tables

**Table 1:** Clinical characteristics of patients with glioblastoma.

Code	Sex	Age	Diagnosis	IDH1	1p19q	TERT	MGMT
GBO21-07	M	64	glioblastoma	wild-type	intact	mutant	unmethylated
GBO21-08	F	85	gliosarcoma	wild-type	intact	mutant	unmethylated
GBO22-16	M	73	glioblastoma	wild-type	intact	mutant	unmethylated
GBO23-01	M	14	glioblastoma	wild-type	intact	wild-type	unmethylated

## Data Availability

The original contributions presented in this study are included in the article/Supplementary Material. Further inquiries can be directed to the corresponding author(s).

## Supplementary Materials

The following supporting information can be downloaded at: https://www.mdpi.com/article/10.3390/cells15171620/s1. Figure S1: Histological and functional characterization of four GBOs; Figure S2: CD::UPRT/5-FC-dependent cytotoxic effects of BM03 in GBOs; Figure S3: Uncropped full-length immunoblot images corresponding to Figure 3C; Figure S4: Uncropped full-length immunoblot images corresponding to Figure 4B; Figure S5: Uncropped full-length immunoblot images corresponding to Figure 5E; Movie S1: Time-lapse imaging of MSC-WT and BM03 migration toward GBOs; Table S1: Information of antibodies used in the study.

## Abbreviations

The following abbreviations are used in this manuscript:

5-FC	5-fluorocytosine
5-FU	5-fluorouracil
BBB	blood–brain barrier
BF	bright-field
BSA	bovine serum albumin
CCK-8	cell Counting Kit-8
CD::UPRT	cytosine deaminase::uracil phosphoribosyltransferase
CNS	central nervous system
DMEM	Dulbecco’s Modified Eagle Medium
DMSO	dimethyl sulfoxide
EMT	epithelial–mesenchymal transition
FBS	fetal bovine serum
GBM	glioblastoma
GBO	GBM patient-derived organoids
H&E	hematoxylin–eosin
IF	immunofluorescence
MSC	mesenchymal stem cells
PBS	phosphate-buffered saline
PI	propidium iodide
PVDF	polyvinylidene difluoride
TBS	Tris-buffered saline
TMZ	Temozolomide
TRAIL	TNF-related apoptosis-inducing ligand
WT	Wild type
WHO	World Health Organization
