# Supplementary material for: Engineered Mesenchymal Stem Cells Expressing CD::UPRT and TRAIL Exhibit Potent Anti-Tumor Effects in Glioblastoma Patient-Derived Organoids
Source: Cells. 2026 Sep 6;15(17):1620. doi: 10.3390/cells15171620 (PMC13565699; doi:10.3390/cells15171620)
Supplement: Supplementary file 1 [file cells-15-01620-s001.zip › Supplementary Material.pdf]

Supplementary Material

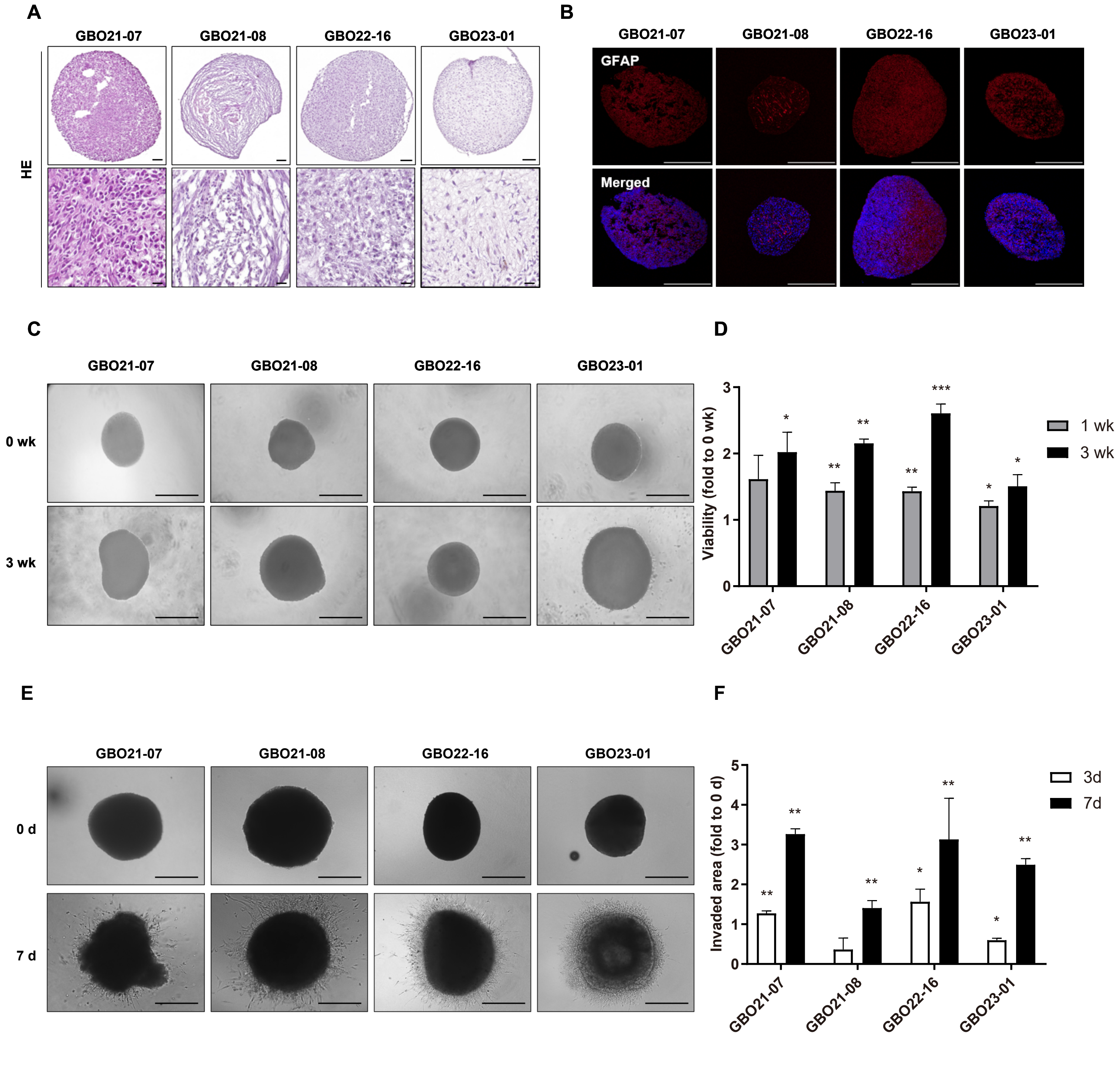

Figure S1. Histological and functional characterization of four GBOs. (**A**) Representative hematoxylin and eosin (H&E)-stained sections of GBO21-07, GBO21-08, GBO22-16, and GBO23-01. Upper and lower panels show low (×8) and high (×40) magnification images, respectively. Scale bars = 100 µm (upper panels) and 20 µm (lower panels). (**B**) Representative immunofluorescence images showing GFAP expression (red) in the four GBO models. Nuclei were counterstained with Hoechst (blue). Scale bar = 500 µm. (**C**) Representative bright-field images of the four GBO models at baseline (0 week) and after 3 weeks of culture. Scale bar = 500 µm. (**D**) Longitudinal viability of the four GBO models assessed by WST assay (n = 5 organoids per GBO model). Viability at 1 and 3 weeks was normalized to the corresponding baseline value at 0 week for each individual GBO and expressed as fold change. Statistical significance was determined using repeated-measures one-way ANOVA followed by Dunnett's multiple comparisons test. Data are presented as mean ± SEM ($^{*}p < 0.05$, $^{**}p < 0.01$, and $^{***}p < 0.001$). (**E**) Representative BF images of the 3D invasion assay at 0 and 7 days, showing invasive outgrowth from the four GBOs. Scale bar = 500 µm. (**F**) Quantification of the invaded area of the four GBO models (n = 3 organoids per GBO model). The invaded area at days 3 and 7 was normalized to the corresponding area at day 0 for each individual GBO and expressed as fold change. Statistical significance was determined using repeated-measures one-way ANOVA followed by Dunnett's multiple comparisons test. Data are presented as mean ± SEM ($^{*}p < 0.05$ and $^{**}p < 0.01$).

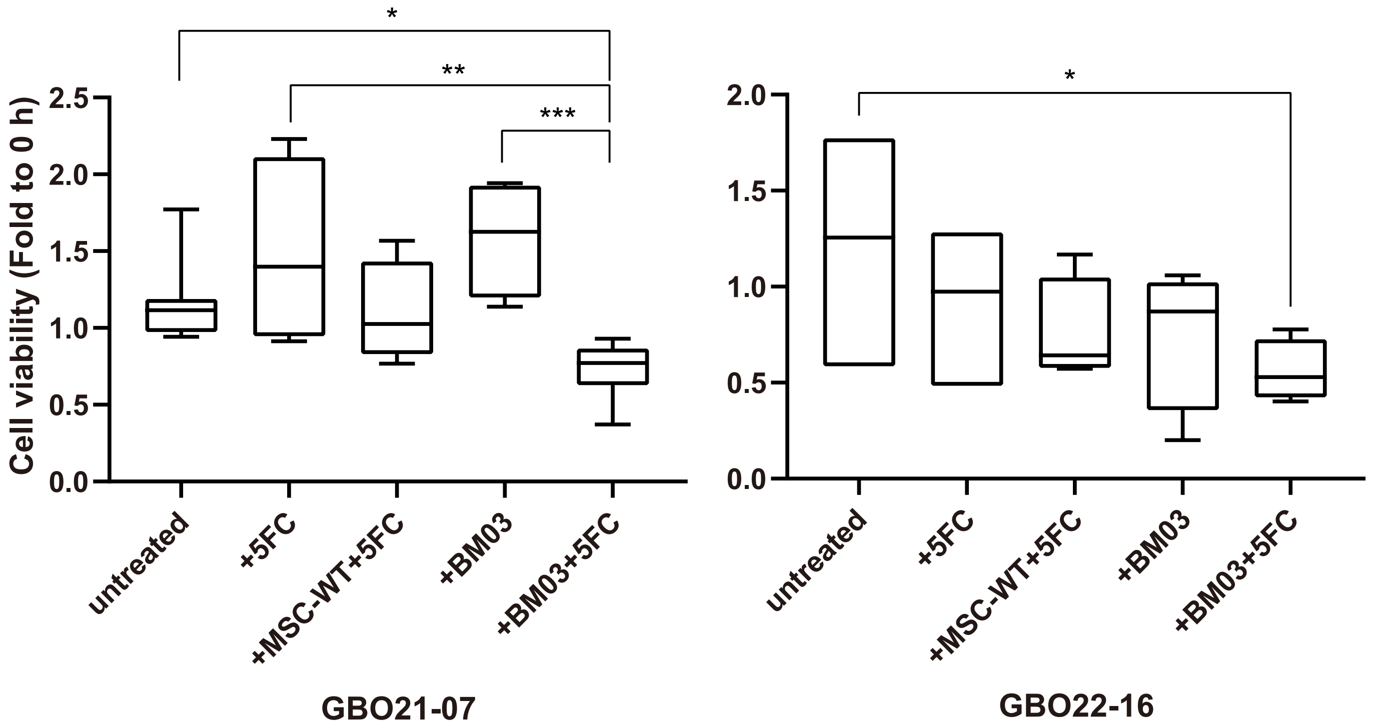

Figure S2. CD::UPRT/5-FC-dependent cytotoxic effects of BM03 in GBOs. Cell viability of (**A**) GBO21-07 (n = 4 per group) and (**B**) GBO22-16 (n = 3 per group) was assessed by WST assay after 72 h under the indicated conditions: untreated, 5-FC alone, MSC-WT + 5-FC, BM03 alone, and BM03 + 5-FC. Viability was expressed as fold change relative to the corresponding 0 h value. Statistical significance was determined by one-way ANOVA followed by Tukey's multiple comparisons test (*$p < 0.05$, **$p < 0.01$, and ***$p < 0.001$).

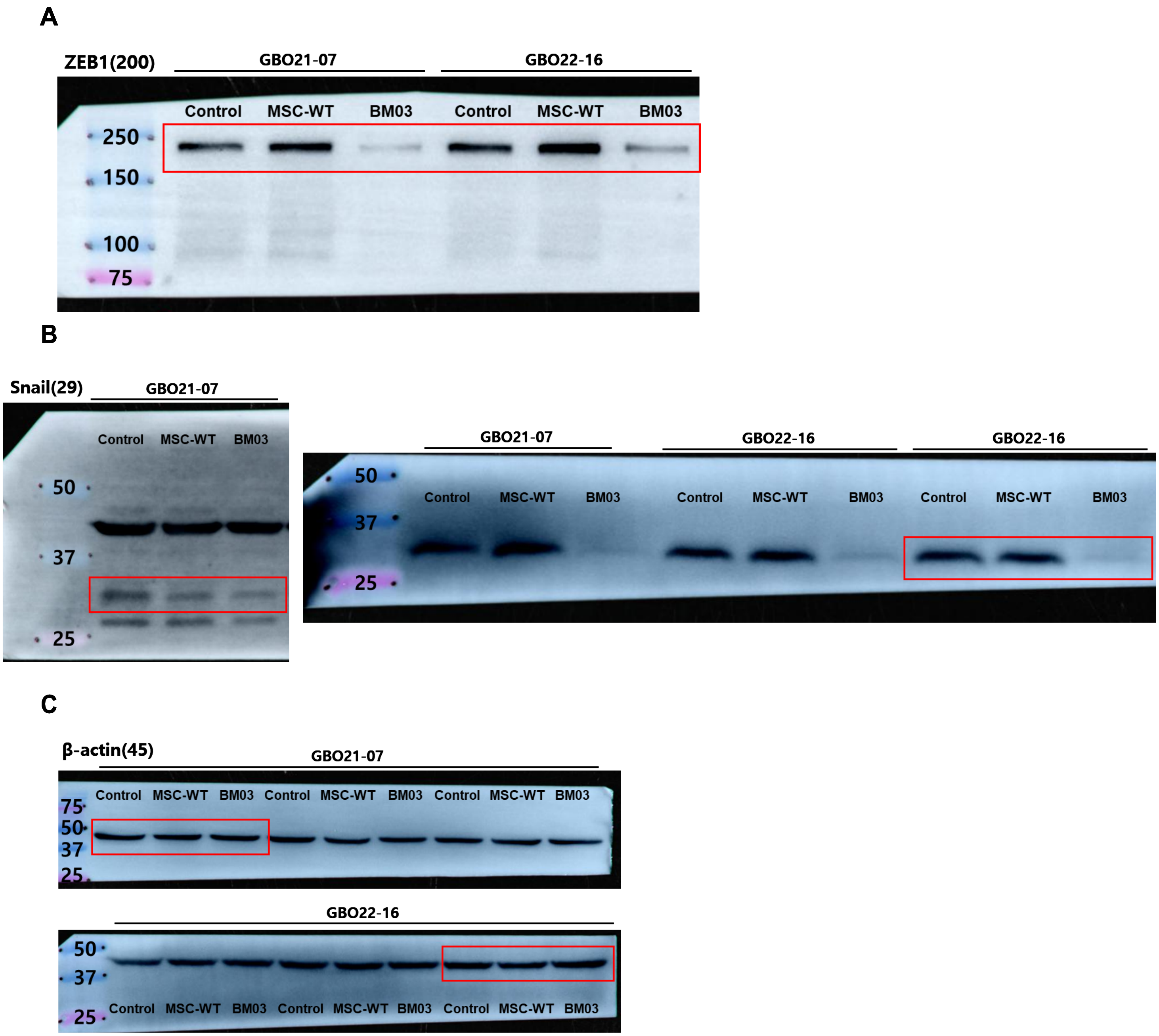

Figure S3. Uncropped full-length immunoblot images corresponding to Figure 3C. Uncropped full-length western blot membrane images showing Zeb1 (**A**), Snail (**B**), and β-actin (**C**) expression in GBO21-07 and GBO22-16. The red boxed regions indicate the bands cropped and shown in Figure 3C. The molecular weight markers, including the corresponding kDa values, are indicated on each membrane. β-actin was used as the loading control.

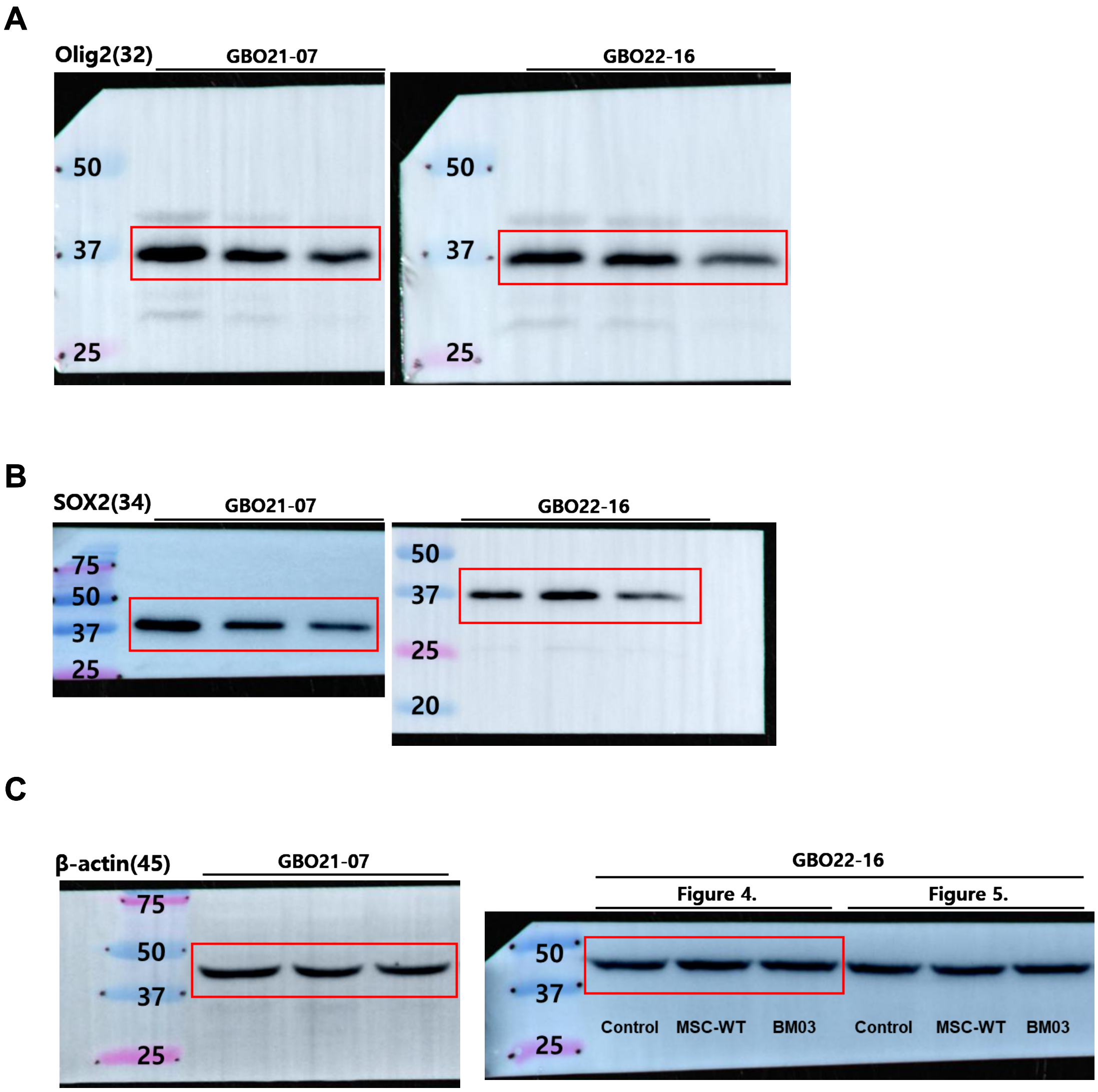

Figure S4. Uncropped full-length immunoblot images corresponding to Figure 4B. Uncropped full-length western blot membrane images showing Olig2 (**A**), Sox2 (**B**), and β-actin (**C**) expression in GBO21-07 and GBO22-16. The red boxed regions indicate the bands cropped and shown in Figure 4B. The molecular weight markers, including the corresponding kDa values, are indicated on each membrane. β-actin was used as the loading control.

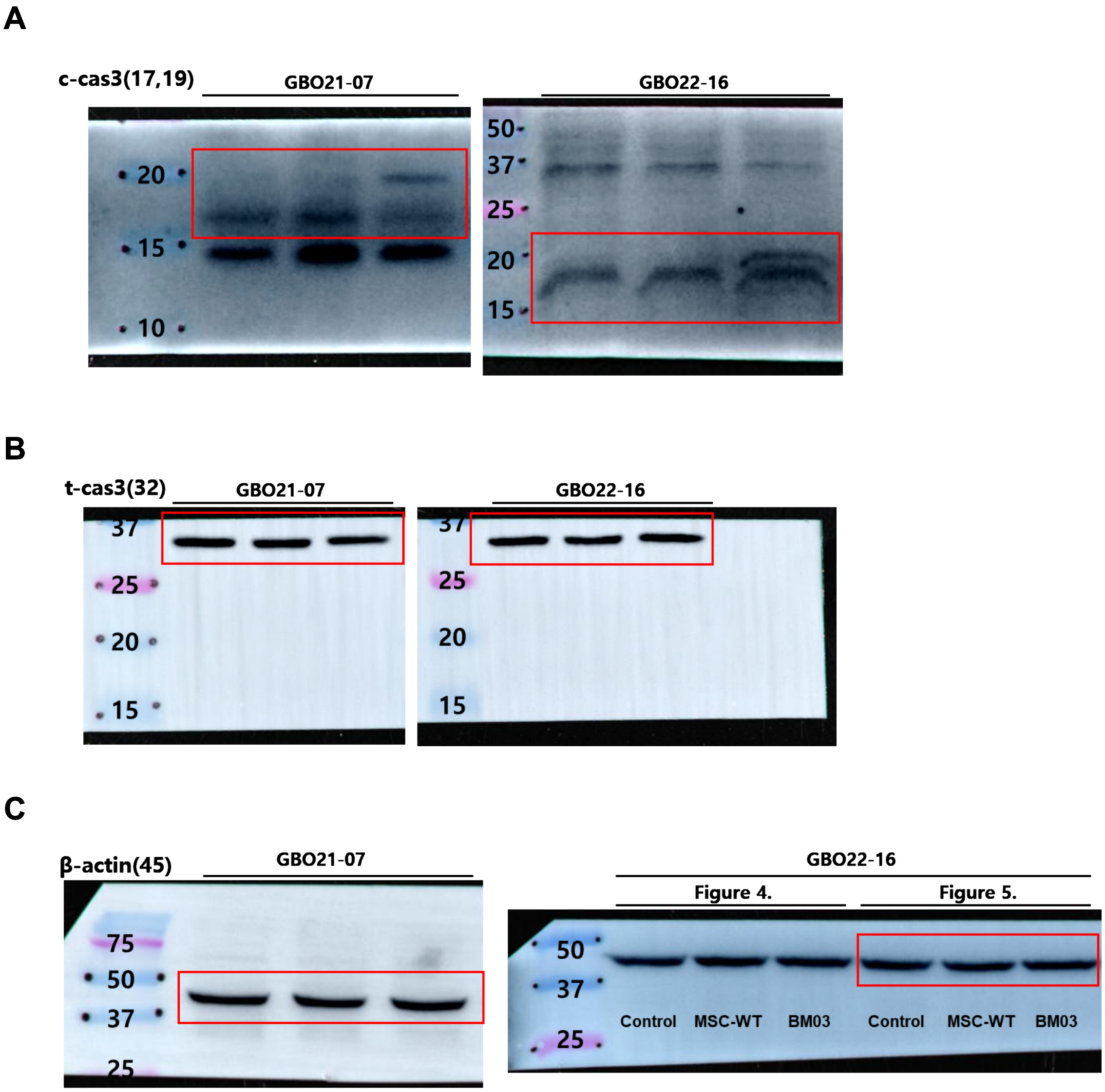

Figure S5. Uncropped full-length immunoblot images corresponding to Figure 5E. Uncropped full-length western blot membrane images showing cleaved caspase-3 (c-cas3; **A**), total-caspase 3 (t-cas3; **B**), and β-actin (**C**) expression in GBO21-07 and GBO22-16. The red boxed regions indicate the bands cropped and shown in Figure 5B. The molecular weight markers, including the corresponding kDa values, are indicated on each membrane. β-actin was used as the loading control.

Movie S1. Time-lapse imaging of MSC-WT and BM03 migration toward GBOs. Time-lapse fluorescence images showing CMFDA-labeled BM03 (**A**) and MSC-WT (**B**) cells migrating toward Hoechst-labeled GBO21-07, CMFDA-labeled BM03 (**C**) and MSC-WT (**D**) cells migrating toward Hoechst-labeled GBO22-16. Images were acquired at 1 h intervals for 12 h. Scale bar, 1000 μm.

Table S1. The information of antibodies used in the study

**Primary antibodies**	**Application**	**Dilution**	**Source**	**Cat no.**
Rabbit monoclonal anti-zeb1	WB	1:1000	Cell Signaling Technology	70512S
Rabbit monoclonal anti-snail	WB	1:1000	Cell Signaling Technology	3879S
Mouse monoclonal anti-GFAP	IF	1:700	Cell Signaling Technology	3670S
Rabbit monoclonal anti-olig2	IF/WB	1:300/1:2000	Abcam	ab109186
Rabbit polyclonal anti-sox2	WB	1:1000	Abcam	ab97959
Rabbit monoclonal anti-cleaved caspase-3	IF/WB	1:500/1:500	Cell Signaling Technology	9664s
Rabbit polyclonal anti-caspase-3	WB	1:1000	Santa Cruz	sc-7148
Rabbit polyclonal anti-β-actin	WB	1:4000	Cell Signaling Technology	4967s

**Secondary antibodies**	**Application**	**Dilution**	**Source**	**Catalog**
goat anti-rabbit IgG-CFL 647	IF	1:500	Santa Cruz	sc-362292
m-IgGκ BP-FITC	IF	1:500	Santa Cruz	sc-516140
mouse anti-rabbit IgG-HRP	WB	1:4000	Santa Cruz	sc-2357
m-IgGκ BP-HRP	WB	1:4000	Santa Cruz	sc-516102
